# The Host Adaptation of *Staphylococcus aureus* to Farmed Ruminants in New Zealand, With Special Reference to Clonal Complex 1

**DOI:** 10.1111/1758-2229.70087

**Published:** 2025-05-06

**Authors:** Jabin Nesaraj, Alex Grinberg, Richard Laven, Ryan Chanyi, Eric Altermann, Claudio Bandi, Patrick J. Biggs

**Affiliations:** ^1^ School of Veterinary Science Massey University Palmerston North New Zealand; ^2^ Blue Barn Life Sciences Ltd. Feilding New Zealand; ^3^ Department of Biosciences University of Milan Milano Italy; ^4^ School of Food Technology and Natural Sciences Massey University Palmerston North New Zealand

**Keywords:** bovine mastitis, comparative genomics, host adaptation, *Staphylococcus aureus*, whole genome sequencing

## Abstract

Genetic features of host adaptation of *S. aureus* to ruminants have been extensively studied, but the extent to which this adaptation occurs in nature remains unknown. In New Zealand, clonal complex 1 (CC1) is among the most common lineages in humans and the dominant lineage in cattle, enabling between‐, and within‐CC genomic comparisons of epidemiologically cohesive samples of isolates. We assessed the following genomic benchmarks of host adaptation to ruminants in 277 *S. aureus* from cattle, small ruminants, humans, and pets: 1, phylogenetic clustering of ruminant strains; 2, abundance of homo‐specific ruminant‐adaptive factors, and 3, scarcity of heterospecific factors. The genomic comparisons were complemented by comparative analyses of the metabolism of carbon sources that abound in ruminant milk. We identified features fulfilling the three benchmarks in virtually all ruminant isolates, including CC1. Data suggest the virulomes adapt to the ruminant niche sensu lato accross CCs. CC1 forms a ruminant‐adapted clade that appears better equipped to utilise milk carbon sources than human CC1. Strain flow across the human–ruminant interface appears to only occur occasionally. Taken together, the results suggest a specialisation, rather than mere adaptation, clarifying why zoonotic and zoo‐anthroponotic *S. aureus* transmission between ruminants and humans has hardly ever been reported.

## Graphical abstract

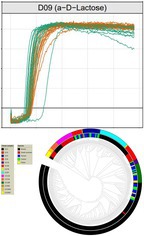

## 1 Introduction

*Staphylococcus aureus* colonises the cornified epithelia of ~30% of humans and is found as a commensal in a range of mammals, reptiles and birds (Haag et al. 2019). As a pathogen, it is a common cause of skin and soft tissue and internal organ infections in humans and several animal species, and endemic, economically important intramammary infections in farmed ruminants. The bacterium has a clonal population structure, with clonal lineages defined by multilocus sequence typing as ‘sequence types’ (STs) that group into broader phylogenetic groups dubbed ‘clonal complexes’ (CCs) (Feil et al. 2003; Hanage et al. 2006; Méric et al. 2015). *S. aureus* strains exchange genetic material through recombination and horizontal gene transfer (Lindsay 2010), leading to substantial differences in gene sequence and content between and within CCs, with a gene flow that is insufficient to blur the genetic distinctiveness of the CCs in most cases (Feil et al. 2003).

Historical host switches of *S. aureus* among humans and ruminants have been inferred from phylogenetic studies (Howden et al. 2023). Yet, direct transmission between these hosts has been rarely documented (Grinberg et al. 2004; Juhász‐Kaszanyitzky et al. 2007; Straub et al. 2024). Hence, ruminant *S. aureus* mastitis is not considered zoonotic, nor is the presence of infected farm personnel deemed a risk factor for mastitis. Studies have indicated that most intramammary infections in farmed ruminants are caused by a limited number of CC/STs, with little overlap with the CC/STs found in humans (Smith et al. 2005; Fitzgerald 2012; Shepheard et al. 2013). While most infections in ruminants have been attributed to lineages belonging to CC97 and CC133, and to ST522, ST151 and ST479, the most common lineages reported in humans are CC1, CC5, CC8, CC12 and CC15 (Smith et al. 2005; Fitzgerald 2012; Schlotter et al. 2012; Shepheard et al. 2013; Boss et al. 2016; Richardson et al. 2018; Haag et al. 2019; Park et al. 2022; Chakrawarti et al. 2024; Gharaibeh et al. 2024). This split in the clonal repertoire motivated the use of terms such as ‘ruminant‐associated’, or ‘ruminant‐adapted’ CC/ST (Park et al. 2010). The clonal split also hindered the study of the adaptation of *S. aureus* to ruminants, due to the difficulty of differentiating host‐adaptive genetic variation, from variation determined by the independent evolutionary history of each CC. Hence, most of what is known is gene‐centric, obtained from surveys of the prevalence of ruminant‐adaptive factors in ruminant‐associated CC/STs, which often yielded variable results (Herron‐Olson et al. 2007; Guinane et al. 2010; Schlotter et al. 2012; Bacigalupe et al. 2019; Matuszewska et al. 2020; Park et al. 2022; Naushad et al. 2020; Chaguza et al. 2022; Keinprecht et al. 2024). To further complicate the picture, in recent years, ‘ruminant‐associated’ CCs have been increasingly isolated from humans, and non‐ruminant associated from ruminants (Bar‐Gal et al. 2015; Chaguza et al. 2022; Nesaraj et al. 2023; White et al. 2024).

The epidemiological situation in New Zealand differs substantially from the rest of the world. Here, CC1 (in particular ST1), an archetypal generalist with no identified host adaptive characteristics (Sung et al. 2008; Shepheard et al. 2013; Grinberg et al. 2017), is the most common CC in humans and the dominant CC in dairy cattle, accounting for about 70% of the infections and circulating alongside typical ruminant‐associated CCs such as CC97 and CC151 (Heffernan et al. 2015; Nesaraj et al. 2023). This situation allowed us to perform comparative genomic analyses of epidemiologically cohesive samples of isolates from different host species, between and (most importantly) within CCs.

We aimed to ascertain the prevalence of host adaptation to ruminants in CC1 and in other CCs, and improve our understanding of the mechanisms of host adaptation, through genome‐wide comparative analyses of quasi‐contemporaneous New Zealand bovine (*n* = 188), small ruminant (*n* = 30), and human, canine and feline (*n* = 59) isolates. Our analysis assessed three genomic benchmarks for bacterial host adaptation (modified from Shepheard et al. 2013; Matuszewska et al. 2020): 1, the presence of phylogenetic clustering; 2, an abundance of accessory homo‐specific (ruminant)‐adaptive genes; and 3, absence or scarceness of accessory heterospecific‐adaptive genes in ruminant isolates. The genomic comparisons were complemented by a comparative phenotypic analysis of the metabolism of lactose and other carbon sources that abound in milk within CC1.

## 2 Materials and Methods

### 2.1 *S. aureus* Isolates and Genome Sequences

We used draft genomes of 277 quasi‐contemporaneous New Zealand methicillin‐susceptible *S. aureus* (MSSA) isolates from dairy cattle (*n* = 188), non‐dairy sheep (*n* = 29), a goat (*n* = 1), humans (*n* = 34), dogs (*n* = 19) and cats (*n* = 6). The bovine isolates were obtained from intramammary infections identified during three time periods between 2002 and 2019, on more than 65 dairy farms on both the North and South Island. These represented the full set of isolates analysed by us in a previous genomic survey (Nesaraj et al. 2023). The previous study revealed no genomic segregations based on the geographical location and collection period, suggesting a stable population in space and time. Additional details on the bovine sample are provided in Supporting Information S1. The sample from small ruminants included 30 ovine isolates collected in 2017–2018 during a survey conducted on 11 North Island non‐dairy sheep farms (Ridler et al. 2021; Supporting Information S1). One other ovine and a goat isolate were isolated by animal diagnostic laboratories in 2018 and 2012, respectively. The genomes of two ovine isolates were discarded due to poor assembly quality, leaving 30 genomes for analysis.

The genomes of human, canine and feline *S. aureus* were from isolates obtained between 2012 and 2016 from nasal colonisation and infection sites, and have been previously analysed (Grinberg et al. 2017; Supporting Information S1). The previous study revealed no genomic segregations based on host species. The isolates used in the present study were all the isolates sequenced in that study, which belonged to five out of the six most common CCs reported in humans in a national survey from 2014 (CC1, CC188, CC5, CC30 and CC15; Heffernan et al. 2015). Additional data used for comparison included the spa‐types of > 700 human *S. aureus* isolates determined in the national survey (Heffernan et al. 2015).

Genome sequencing, assembly and annotation of the bovine and human/canine/feline isolates have been described in the original papers. Sequencing of the small ruminant isolates was performed ex novo at the Centro di Ricerca Pediatrica Romeo ed Enrica Invernizzi, University of Milan, Italy. Briefly, DNA libraries were prepared using the Nextera XT library preparation kit (Illumina, San Diego, USA) and sequenced on a MiSeq with 2 × 250 bp paired‐end (Illumina, San Diego, USA). Genome assembly and annotation used the same methods applied to the bovine isolates (Nesaraj et al. 2023).

## 3 Bioinformatic Analysis

### 3.1 Determination of the STs, CCs and Spa‐Types

The STs of the isolates were defined using the method described by Nesaraj et al. (2023). Briefly, an online mlst tool was used to extract the alleles from the contigs (https://github.com/tseemann/mlst). STs were assigned to CCs in PubMLST (Jolley et al. 2018), which uses the BURST algorithm (Feil et al. 2004). Groups of single‐locus variants (SLV) or double‐locus variants (DLV) that could not be assigned to one of the nine CCs defined in PubMLST as of February 2021 were defined as a CC, which name took the number of the central ST in the group (i.e., the ST with the greatest number of SLV in the group). Any new allele not reported in PubMLST was mapped against all the reads using samtools (Li et al. 2009) to verify that the new allele sequence was not due to assembly errors. After verification, novel STs were submitted to the PubMLST database, where a new ST identifier was assigned by the database curators. For comparative purposes, the six commonest CCs identified in humans in New Zealand during a previously published national survey were also tabulated (Heffernan et al. 2015).

Spa‐typing is a typing method based on the analysis of sequence variation in a variable repeat region of the Staphylococcal protein A (Spa) gene. In this study, the spa‐types were defined using the tool ‘spa‐typing’ (https://github.com/mjsull/spa_typing) that identifies the repeat sequences from the contigs and defines the Spa‐type using the Ridom database (http://www.ridom.de/). Any Spa‐type not able to be identified in the Ridom database was assigned with ‘t‐new’.

### 3.2 Assessment of Phylogenetic Clustering of Ruminant *S. aureus* (Benchmark 1)

This analysis pertained to the core genome, the stable genomic fraction that accumulates vertical genetic variation. As we analysed draft genomes, our working definition of ‘core genome’ was the complete set of genes identified in all the isolates. These genes were determined from the annotated files using Roary 3.11.2 (Page et al. 2015), as previously described (Nesaraj et al. 2023). The core gene sequences of all the isolates were concatenated and aligned using Roary. To assess for phylogenetic clustering, we used the nucleotide core genome pairwise distance matrix to construct a Jukes‐Cantor neighbor‐joining tree (NJ) in Geneious 6.1, with 1000 bootstrap resamplings (http://www.geneious.com/). Additional NJ trees were constructed separately for CC1 and any other CC represented by more than one isolate in each host species. To increase the number of ‘core’ genes contributing to the trees (Grinberg et al. 2017), only one arbitrarily selected ruminant isolate per farm was included in these additional trees.

### 3.3 Assessment of the Presence of Homo‐Specific and Heterospecific Host‐Adaptive Genes in Ruminant Isolates (Benchmarks 2 and 3)

These benchmarks pertained to the accessory genome, which we defined as the set of observed genes that were not present in all the isolates. The analysis began with the visualisation of a principal coordinate analysis plot (PCoA) derived from the Roary gene presence–absence matrix, and implemented in PAST software (Hammer et al. 2001). Before the PCoA, clonal replicates were eliminated by including in the analysis only one arbitrarily selected ruminant isolate per CC/farm combination, which reduced the dataset to 212 isolates. A second, more targeted PCoA used the gene presence–absence matrix of the virulome generated by the programme ARIBA (Hunt et al. 2017), which mapped the reads to the virulence factor sequences downloaded from the Virulence Factors Database (VFDB) (Chen et al. 2005).

The PCoA was followed by gene‐by‐gene searches targeting mobile genes coding for known ruminant and human host‐adaptive factors reviewed by Matuszewska et al. (2020) and presented in Table 1. These genes were searched in the ARIBA gene presence/absence matrix. The number of isolates carrying each gene was tabulated for comparison. In addition, antimicrobial resistance (AMR) genes were searched using ARIBA, which mapped the reads to the reference AMR gene sequences downloaded from the online database Resfinder (Bortolaia et al. 2020).

**TABLE 1:** Host adaptive factors searched in the *S. aureus* genomes in this study.

Host‐association	Gene	Protein coded	Host adaptive function
Humans	*scn*	Staphylococcal complement inhibitor	Blocks the activation of the human complement system (van Wamel et al. 2006; Richardson et al. 2018)
	*sak*	Staphylokinase	Converts human plasminogen to plasmin (van Wamel et al. 2006; Richardson et al. 2018)
	*chp*	Chemotaxis inhibitory proteins of staphylococci	Inhibits the infiltration of human neutrophils (unknown functionality in ruminants) (van Wamel et al. 2006; Richardson et al. 2018)
	*sea*	Staphylococcal enterotoxin A (SEA)	Human superantigen (van Wamel et al. 2006; Richardson et al. 2018)
	*lukS‐PV* and *lukF‐PV*	Panton Valentine leukocidin (PVL)	Lysis of human neutrophils (does not produce lysis of bovine neutrophiles) (Melles et al. 2006)
Ruminants	*tsst*	Toxic shock syndrome toxin (TSST)	Activates bovine T cells (Fitzgerald et al. 2001; Wilson et al. 2018)
	*sec*	Staphylococcal enterotoxin C (SEC)	Superantigen associated with bovine strains (Fitzgerald et al. 2001)
	*sel*	Staphylococcal enterotoxin L (SEL)	Superantigen associated with bovine strains (Orwin et al. 2003)
	*lukM* and *lukF′*	Leukocidin	Lysis of ruminant neutrophils (does not produce lysis of human neutrophils; Vrieling, Koymans, et al. 2015; Vrieling, Boerhout, et al. 2016)

## 4 Comparative Analysis of the Metabolism of Carbon Sources Abundant in Ruminant Milk

We sought to assess if ruminant CC1 utilise lactose and other carbon sources available in bovine milk more effectively than human strains, conforming to a previous study on ST97 (Richardson et al. 2018). To do this, we selected at random 10 bovine CC1 and six human CC1/ST1 for a comparison of their ability to utilise four milk carbon sources (D‐lactose, D‐galactose, butyric acid and caproic acid) included in the PM1 and PM2 plates of the Omnilog Phenotype Microarray (USA) system. D‐lactose and D‐galactose are the principal carbohydrates of milk, and butyric acid and caproic acid are highly specific products of ruminal microbial activity present in bovine milk (Månsson 2008). The 10 bovine CC1 isolates were chosen at random from the collection, to include one isolate per farm. The human isolates were selected from skin and soft tissue infection (SSTI) cases (see Grinberg et al. 2017; Supporting Information S1). The Omnilog measures colour change of a thioredoxin dye due to NADH produced by the bacteria in the presence of each single carbon source to calculate an ‘Omnilog value’. The plates were prepared according to the manufacturer's protocol for Gram‐positive bacteria with modification (Supporting Information S2). The 16 isolates were run in duplicates for a total of 32 determinations. A blank without carbon source was included for each isolate to validate the procedure. Plates were incubated at 37°C, with optical measurements taken at 15‐min intervals for 48 h. The Omnilog kinetic growth curves (i.e., the plots of the Omnilog values obtained every 15 min over 48 h) for the four compounds were visualised. For completeness, the sequences of the eight genes composing the *S. aureus* lactose operon (*lac*A, B, C, D, F, E, G, R) were extracted from all the CC1 isolates, to assess for systematic amino acid variation between ruminant and human/canine/feline CC1 strains.

## 5 Results

### 5.1 Distribution of CCs, STs and Spa‐Types

Table 2 shows the distribution of CCs and STs across the host species. As reported in the original papers, CC1 was the dominant CC in cattle (74%) and the second most common in the human/canine/feline isolates. ST1 was the most common ST within CC1. A total of 15 novel STs were identified and assigned unique ST identifiers by PubMLST. One novel ST, ST5367, was identified in bovine (2/188; 1.1%) and small ruminant (3/30; 10%) isolates (it is reported as CC5367 as it did not have SLVs or DLVs). There was broad sharing of CCs between bovine and small ruminant isolates and—as reported in the original paper—between human, canine and feline isolates. However, apart from CC1, overlaps of CCs between ruminants and non‐ruminants were sporadic. This included CC5, the most common CC among human/canine/feline isolates (25%), which was represented by only one bovine isolate, and C15, observed in three human/canine/feline and one bovine isolate. The second commonest CC was CC97 (9.7%), represented only in cattle. The third was CC8, represented in 3% of bovine and 27% of small ruminant isolates. CC133 was also represented in bovine and small ruminants. One of the most common CCs reported in the New Zealand survey from 2014 (5.8%) was CC121, but it was not observed in our study.

**TABLE 2:** Distribution of clonal complexes and sequence types across host species among 277 *S. aureus* isolates. The right column reports the main CCs found in humans in the national survey from 2014 (Heffernan et al. 2015).

Clonal complex	Sequence types	Total isolates (*n* = 277)	Bovine (*n* = 188)	Small ruminant (*n* = 30)	Human, canine and feline (*n* = 59)	Humans in New Zealand (in percent)
CC1	ST1	149 (53.8%)	133 (71%)	0	16 (27.1%)	
	ST3615	1 (0.4%)	0	1 (3.3%)	0	
	ST4551	1 (0.4%)	1 (0.5%)	0	0	
	ST6140	2 (0.7%)	2 (1.1%)	0	0	
	ST6141	2 (0.7%)	2 (1.1%)	0	0	
	ST6161	1 (0.4%)	1 (0.5%)	0	0	
	ST6163	1 (0.4%)	1 (0.5%)	0	0	
	Total CC1	157 (56.7%)	140 (74%)	1 (3.3%)	16 (27.1%)	15.3%
CC97	ST97	16 (5.8%)	16 (8.5%)	0	0	
	ST6160	8 (2.9%)	8 (4.3%)	0	0	
	ST6162	1 (0.4%)	1 (0.5%)	0	0	
	ST6164	1 (0.4%)	1 (0.5%)	0	0	
	ST71	1 (0.4%)	1 (0.5%)	0	0	
	Total CC97	27 (9.7%)	27 (14.4%)	0	0	
CC151	ST151	6 (2.2%)	6 (3.2%)	0	0	
	ST705	1 (0.4%)	1 (0.5%)	0	0	
	Total CC151	7 (2.5%)	7 (3.7%)	0	0	
CC8	ST8	11 (4.0%)	3 (1.6%)	8 (26.7%)	0	
	ST6143	3 (1.1%)	3 (1.6%)	0	0	
	Total CC8	14 (5.1%)	6 (3.2%)	8 (26.7%)	0	
CC133	ST133	8 (2.9%)	0	8 (26.7%)	0	
	ST701	1 (0.4%)	0	1 (3.3%)	0	
	ST6137	1 (0.4%)	0	1 (3.3%)	0	
	ST6138	1 (0.4%)	0	1 (3.3%)	0	
	ST6139	1 (0.4%)	0	1 (3.3%)	0	
	ST6157	1 (0.4%)	0	1 (3.3%)	0	
	ST6165	1 (0.4%)	1 (0.5%)	0	0	
	ST6166	1 (0.4%)	0	1 (3.3%)	0	
	Total CC133	16 (5.8%)	2 (1.1%)	14 (46.7%)	0	
CC5367	ST5367	5 (1.8%)	2 (1.1%)	3 (5.1%)	0	
CC5	ST5	14 (5.8%)	1 (0.5%)	0	13 (25.4%)	
	ST835	1 (0.4%)	0	0	1 (1.7%)	
	ST1259	1 (0.4%)	0	0	1 (1.7%)	
	Total CC5	16 (5.8%)	1 (0.5%)	0	15 (25.4%)	8.6%
CC45	ST508	1 (0.4%)	1 (0.5%)	0	0	
CC15 (t084)	ST15	2 (0.7%)	0	0	2 (3.4%)	
	ST199	1 (0.4%)	1 (0.5%)	0	0	
	ST582	1 (0.4%)	0	0	1 (1.7%)	
	Total CC15	4 (1.4%)	1 (0.5%)	0	3 (5.1%)	2.8%
CC78	ST78	1 (0.4%)	1 (0.5%)	0	0	
CC30	ST30	5 (2.8%)	0	0	5 (13.5%)	1.9%
	ST34	1 (0.4%)	0	0	1 (1.7%)	
	ST39	1 (0.4%)	0	0	1 (1.7%)	
	ST30v	1 (0.4%)	0	0	1 (1.7%)	
	Total CC30	8 (2.8%)	0	0	8 (13.5%)	
CC692	ST692	1 (0.4%)	0	1 (3.3%)	0	
CC1640	ST1640	3 (1.1%)	0	3 (10.0%)	0	
CC188	ST188	15 (6.1%)	0	0	15 (28.8%)	
	ST188v	2 (0.7%)	0	0	2	
	Total CC188	17 (6.1%)	0	0	17 (28.8%)	10.2%
Other						CC121 (5.8%)

A total of 76 spa‐types were identified (human/canine/feline: 24 spa‐types; ruminants: 54 spa‐types). The distribution of spa‐types also showed very little overlap between ruminants and human/canine/feline isolates. Within CC1, bovine and human/canine/feline isolates only shared s127, and within CC15, they shared t084 (Supporting Information S3). Moreover, only three of the 54 spa‐types represented in ruminants in this study were identified in the New Zealand survey from 2014 (Heffernan et al. 2015).

### 5.2 Ruminant *Staphylococcus aureus* Forms Distinct Phylogenies

**FIGURE 1:**
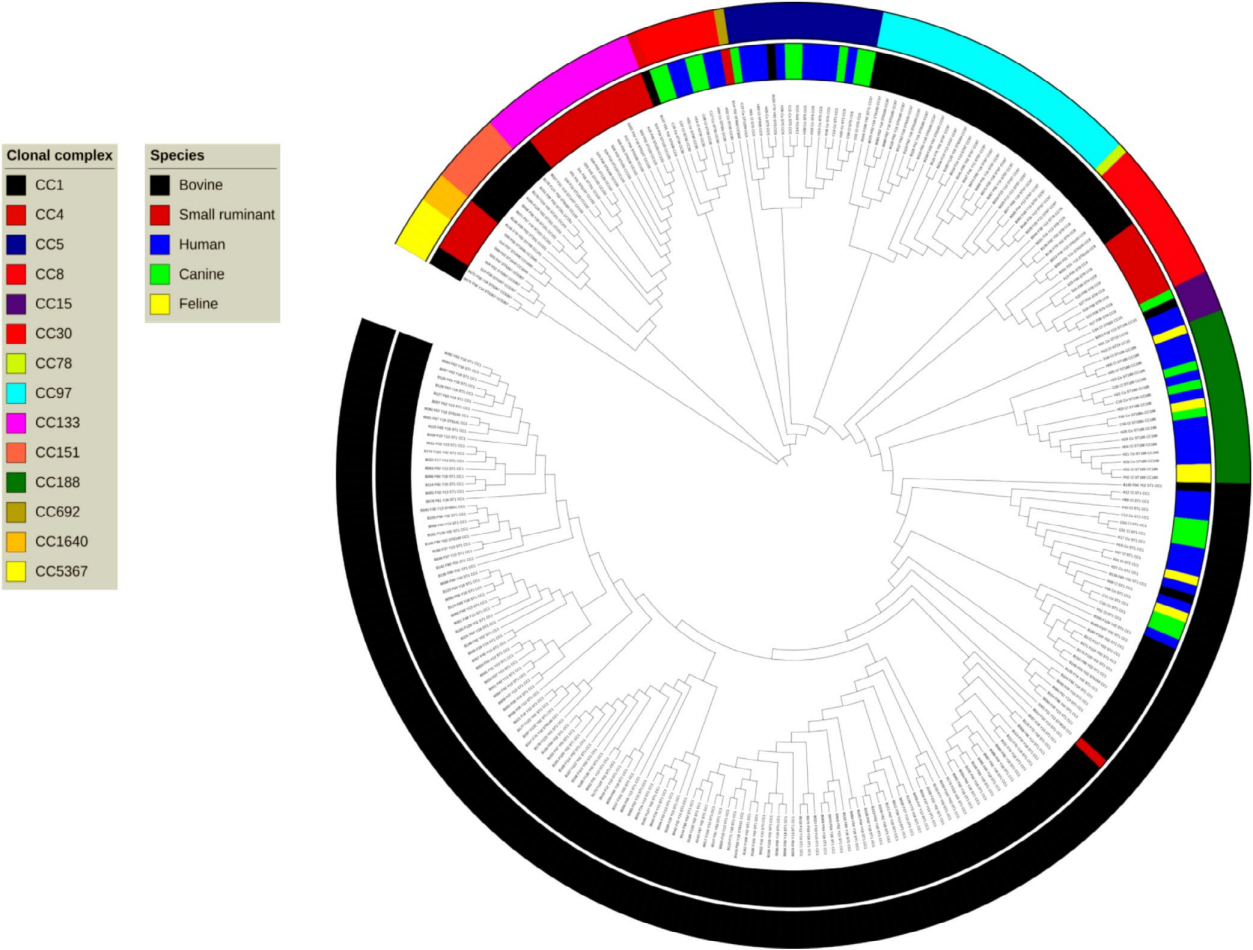
Neighbor‐joining tree of 277 New Zealand *S. aureus* isolates. The outer circle classifies the clonal complexes and the inner circle the host species. The letter ‘F’ in the identifier of bovine and small ruminant isolates is for ‘farm’, and is followed by the numerical farm identifier. The letter ‘Y’ is followed by the year of collection (Nesaraj et al. 2023). ST: sequence type. Note the quasi‐complete separation of ruminant and human/canine/feline CC1, and of bovine and small ruminant CC8 and CC133, and the co‐mingling of human, canine and feline isolates across CCs.

**FIGURE 2:**
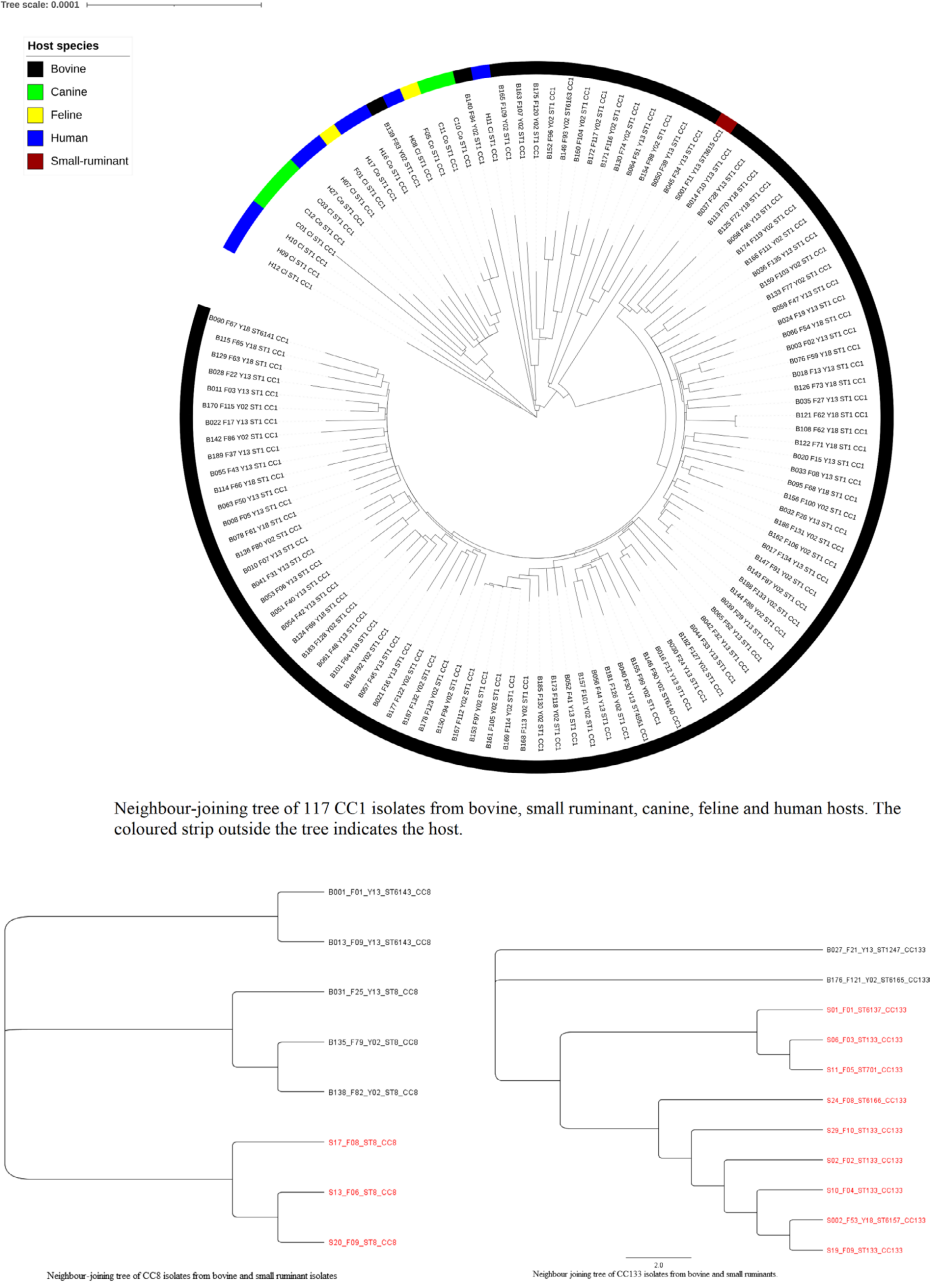
Jukes‐Cantor neighbor‐joining tree of 117 CC1 (upper circular tree), 19 CC133 (middle tree) and eight CC8 isolates (lower tree). In CC133 and CC8, sheep isolates are in red, and bovine isolates are in black. B: bovine; C: canine; F: feline; H: human; S: small ruminant. One isolate per farm was used. Note the segregation according to host species in all three CCs, except for human, canine and feline isolates that co‐mingle. The segregation by ruminant farm can be observed in Figure 1.

The NJ trees are presented in Figures 1 and 2. As expected from a clonal species, each CC formed a monophyletic group. Each host species formed a monophyletic group within each CC, except for the human, canine and feline isolates that co‐mingled in each shared CC, as originally reported (Grinberg et al. 2017). Interestingly, the ruminant CC5 and CC15 did not segregate, but co‐mingled with human/canine/feline isolates.

The segregation between ruminant and human/canine/feline isolates within CC1 was quasi‐complete, with only one bovine (B139) and one human isolate (H11) grouping with the other host, and one bovine isolate (B140) that did not group. Human, canine, and feline isolates co‐mingled without forming monophyletic branches in CC1, CC5, CC30 and CC188, as previously reported (Grinberg et al. 2017). In contrast, within CC133 and CC8, ovine and bovine isolates did not co‐mingle, and each species formed a monophyletic group. A third level of clustering was by farm, with isolates from single bovine and ovine farms forming monophyletic groups in all cases.

### 5.3 The Accessory Genomes of Ruminant *S. aureus* Are Differentiated

**FIGURE 3:**
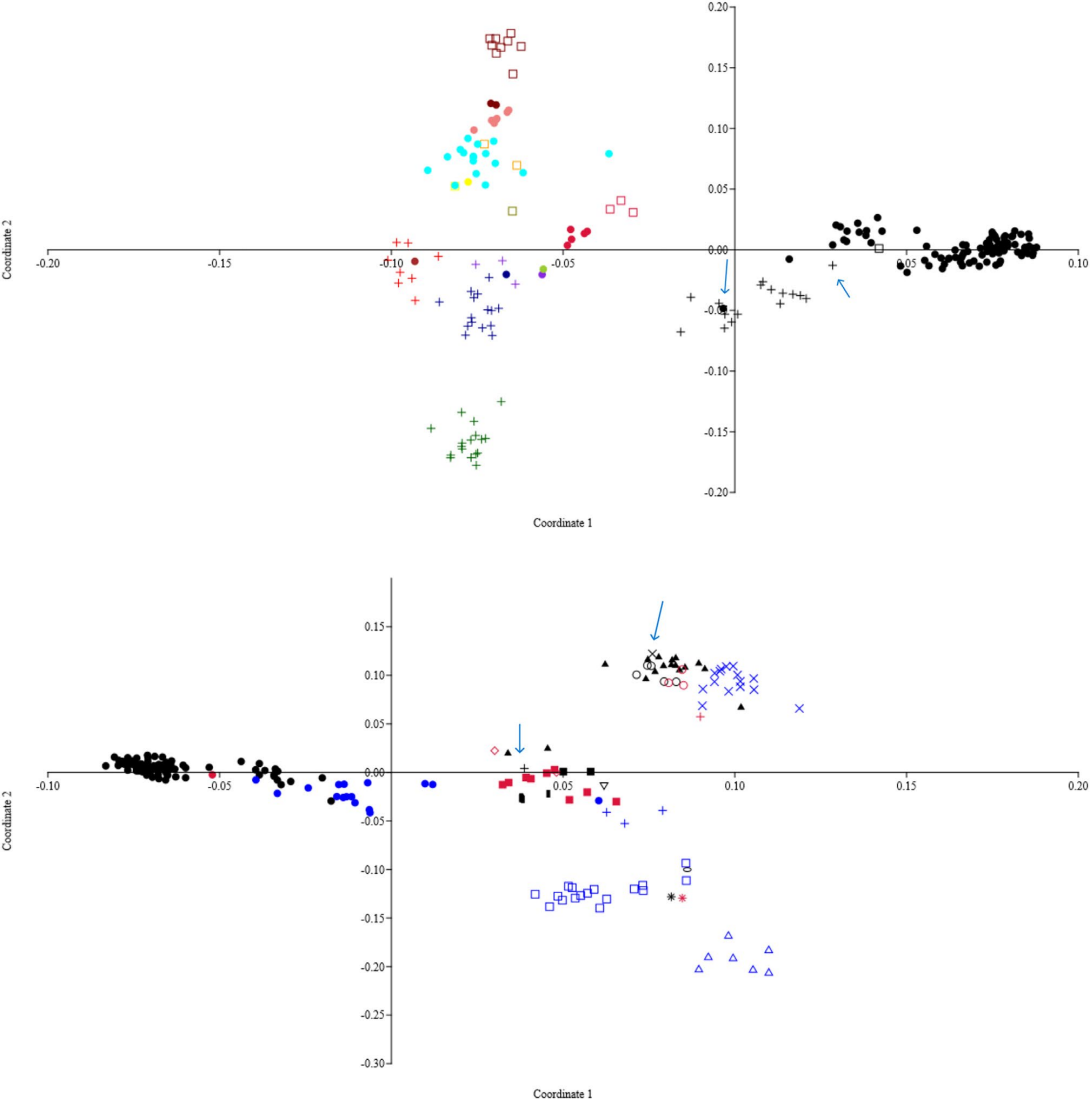
**Upper plot:** principal coordinate analysis (PCoA) plot obtained using the Roary binary gene/absence matrix of 212 *S. aureus* isolates. **Lower plot**: PCoA plot obtained from the ARIBA virulome binary gene/absence matrix of 211 isolates. The two main coordinates explained more than 50% of the variation in both cases. Isolates are represented by data points positioned in the bidimensional space delineated by the two main coordinates. In the upper plot, B139 segregates with human/canine/feline CC1 and H11 segregates with bovine CC1 (blue arrows). In the lower plot, note the bovine CC15 (black plus) and CC5 (black X) segregating from the human CC15 and CC5 and co‐mingling with other ruminant CCs (blue arrows). In the same area, note also co‐mingling of ruminant virulomes from different CCs. For a differentiation between human, canine and feline isolates, see the original paper (Grinberg et al. 2017). **Upper plot:** bovine isolates—dots; human, canine and feline—plus; small ruminants—squares. Black—CC1, brown—CC45, dark blue—CC5, crimson—CC8, blue‐violet—CC15, red—CC30, green—CC78, aqua—CC97, maroon—CC133, light coral—CC151, dark green—CC188, olive—CC692, orange—CC1640, yellow—CC5367. **Lower plot:** bovine isolates—black; human, canine, and feline—blue; small ruminants—red. The isolates are identified by their clonal complexes (CCs): CC1—dots, CC8—'O', CC97—solid triangle, CC15 and CC692—plus, CC133—solid square, CC78—inverted triangle, CC151—bar, CC5367—star, CC5—X, CC45—oval, CC188—square, CC30—triangle, and CC1640—diamond.

The Roary gene presence–absence matrix was used to generate a PCoA plot representing the relationships of the accessory genomes of 212 isolates (Figure 3, upper graph). The primary segregation was according to CC and within each CC based on host species, except the human, canine and feline isolates that co‐mingled in the bidimensional space delineated by the two main coordinates within each CC. Remarkably, B139 and H11 clustered with the other host types also in the PCoA plot. ARIBA identified 197 virulence genes in 211 isolates (one human isolate had failed to run in the ARIBA process), and the PCoA plot generated from this virulome showed a different picture: while CC1 separated from the other CCs, with separation of ruminant from human/canine/feline isolates, segregations of the virulomes of the other CCs were not pronounced, and there was co‐mingling of bovine and ovine virulomes from different CCs (Figure 3, lower graph). Interestingly, the virulomes of the sole ruminant CC15 and CC5 clustered with other ruminant CCs.

### 5.4 Abundance of Homo‐Specific and Scarcity of Hetero‐Specific Host‐Adaptive Factors in Ruminant *S. aureus* , Across the Clonal Landscape

Significant gene–host associations were observed in the sample (Table 3; Supporting Information S4). The genes encoding the bovine‐adaptive bi‐component leukocidin lukF′lukM′, which targets bovine neutrophils (Vrieling, Koymans, et al. 2015), were found in most bovine (66%) and small ruminant (65%) isolates across six CCs, but in no human/canine/feline isolate. Interestingly, the lukM was also found in the sole ruminant CC15. Conversely, genes encoding the human‐adaptive counterpart, the bi‐component Panton Valentine leukocidin (PVL) (Löffler et al. 2010) were found in 6/58 human/canine/feline isolates, but only in one ruminant isolate, the same B139 that clustered with human/canine/feline CC1 in the NJ trees and PCoA.

**TABLE 3:** Distribution of host‐adaptive factors searched in the VFDB database by ARIBA, among 212 *S. aureus* isolates (one isolate per CC/farm combination). The distribution of the *luk*F and *luk*M among CCs is also provided.

Virulence factors	Bovine (*n* = 136)	Human/canine/feline (*n* = 58)	Small ruminants (*n* = 17)
*lukF′*	*n* = 90 (66.2%); (CC1: 80; CC151: 7; CC15: 1; CC97: 2)	0	*n* = 11 (64.7%); (CC133: 9; CC8: 2)
*lukM*	*n* = 89 (65.4%) (CC1: 79; CC151: 7; CC97: 2; CC15: 1)	0	*n* = 12 (70.6%) (CC133: 9; CC8: 3)
*lukF‐PV*	1 (0.7%)	6 (10.3%)	0
*lukS‐PV*	1 (0.7%)	6 (10.3%)	0
*scn*	4 (2.9%)	39 (67.2%)	0
*sak*	4 (2.9%)	49 (84.5%)	0
*chp*	2 (1.5%)	32 (55.2%)	0
*sea*	1 (0.7%)	13 (22.4%)	0
*tsst*	1 (0.7%)	4 (6.9%)	9 (52.9%)
*icaA*	51 (37.5%)	56 (96.6%)	17 (100%)
*icaB*	126 (96.2%)	48 (82.8%)	16 (94.1%)
*icaC*	133 (97.8%)	49 (84.5%)	17 (100%)
*icaD*	49 (36.0%)	57 (98.3%)	17 (100%)
*icaR*	134 (98.5%)	41 (70.7%)	16 (94.1%)

Abbreviations: *chp*, chemotaxis inhibitory protein; *ica*, intercellular adhesion operon containing A, B, C, D and R genes; *lukF‐PV*, F subunit of the Panton Valentine leukocidin; *lukF′*, F subunit of the bovine‐adaptive leukocidin lukMF′; *lukM*, M subunit of the leukocidin lukMF′; *lukS‐PV*, S subunit of the Panton Valentine leukocidin; *sak*, staphylokinase; *scn*, staphylococcal complement inhibitor; *sea*, staphylococcal enterotoxin A; *tsst*, toxic shock syndrome toxin.

Human *S. aureus* strains often carry genes of the human‐adaptive immune‐evasion cluster (IEC) transported in prophage φSaint3, namely *scn* (staphylococcal complement inhibitor), *chp* (chemotaxis inhibitory protein), *sak* (staphylokinase) and *sea* (staphylococcal enterotoxin variant A) (van Wamel et al. 2006; Richardson et al. 2018). In our study, these genes were found in 22% (*sea*) to 84% (*sak*) of human/canine/feline isolates, but only in 1%–3% of bovine isolates, and were not found in any small ruminant isolate. Interestingly, B139 carried *scn* and *sak*. Most human CC5 and CC15 carried *scn*, *sak* and *chp*, but the sole bovine CC5 did not carry any of these genes. The sole bovine CC15 carried *scn*, *sak* and *chp*.

The differential distribution of the other genes was not clear‐cut. Among the staphylococcal enterotoxins, the *sec* variant was found largely among small ruminant isolates belonging to CC133 (9/17; 52.9%). Two (1.5%) bovine isolates had the *sec* variant (CC78 and CC151), and none of the human/canine/feline isolates had this enterotoxin. The toxic shock syndrome toxin gene (*tsst*) was found in one bovine isolate and nine small ruminant C133, and in human/canine/feline CC30.

The resistome appeared unevenly distributed, with human isolates being 2.6 times more likely to carry any AMR gene than ruminant isolates (95% CI: 1.7–3.9) (Supporting Information S5). In particular, *bla*Z (a β‐lactamase) was found in ~70% of human/canine/feline isolates but only in ~23% of ruminant isolates. Other AMR genes were sporadic and included the *ant* (aminoglycoside aminotransferase), *aph* (aminoglycoside phosphotransferase), *ermA*, *ermB* and *ermC* (erythromycin ribosomal methylases), *fusc* (fusidic acid resistance) and *str* (streptomycin resistance) genes.

### 5.5 Bovine and Human CC1 Differ in Their Ability to Utilise Milk Carbon Sources

**FIGURE 4:**
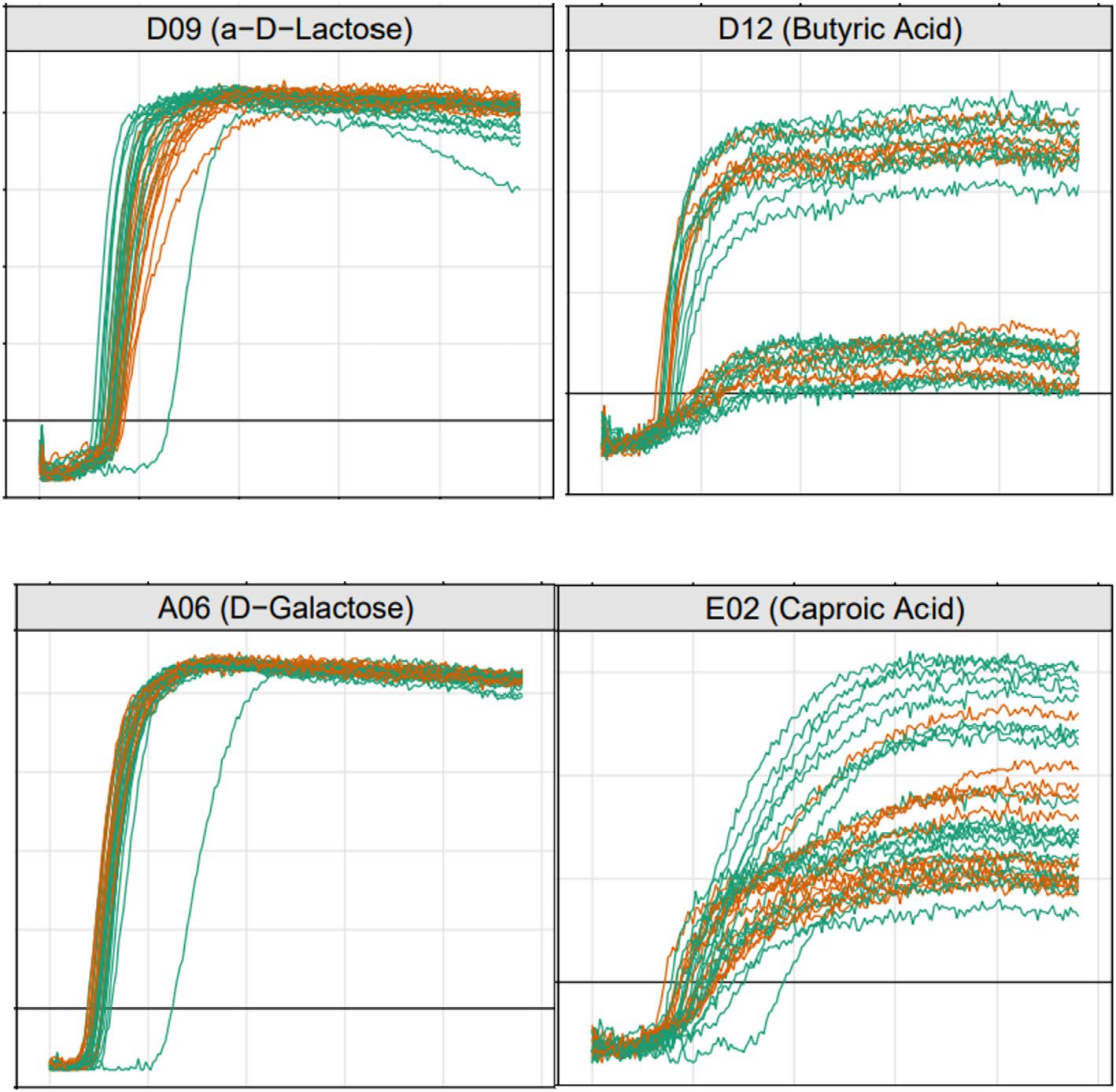
Omnilog kinetic curves of six human and 10 bovine CC1 isolates in the presence of D‐lactose, butyric acid, D‐galactose and caproic acid. Each isolate was tested in duplicate, for a total of 32 curves. *X*‐axis: time; *Y*‐axis: recorded Omnilog value. Bovine isolates are in green and human isolates in light brown. Note the split between bovine and human curves for lactose and caproic acid, and the co‐mingling of the curves for butyric acid and galactose.

The kinetic curves of lactose and caproic acid differentiated between the human and the bovine isolates (Figure 4). In both curves, the Omnilog values of bovine CC1 appeared to peak faster than those of human CC1, suggesting a faster growth of bovine CC1 in the presence of these compounds. Several bovine caproic acid curves showed significantly greater Omnilog value peaks, suggesting a more thorough utilisation of this compound by these strains within the 48 h of incubation. The kinetic curves of galactose and butyric acid did not differentiated between the host types.

The amino acid sequence variability in the lactose operon did not define a regular pattern. In total, 13 amino acid substitutions were identified in the lactose operon. Most CC1 isolates displayed one substitution in a single gene, and one isolate had two substitutions, one in each of two genes. One bovine isolate had a truncated *LacE*.

## 6 Discussion

The global literature indicated a split in the clonal repertoire of *S. aureus* between ruminants and humans, and an association of certain CC/ST with ruminants. In New Zealand, CC1 is the most common CC in humans and the dominant CC in dairy cattle. This unique situation allowed us to perform within‐ and between‐CC comparisons of epidemiologically cohesive samples of isolates from different hosts, contributing in different ways to the discussion on the host adaptation of *S. aureus* to farmed ruminants.

The phylogenetic clustering of ruminant isolates was reflected by the presence of a dominant ruminant clade within CC1, and the circulation of some archetypical ruminant‐associated CCs such as CC97 and CC133, in ruminants only. A limitation of our study was the relatively modest sample size of human/canine/feline MSSA. Hence, we consulted a published database of contemporaneous Spa‐types of > 700 New Zealand human MSSA (Heffernan et al. 2015), and found very little overlap of spa‐types with our ruminant isolates, consistent with the segregation of the core genomes observed in our study. These findings fulfil the first benchmark of host adaptation.

The NJ trees revealed additional levels of clustering. Firstly, bovine and small ruminant isolates were segregated in CC8 and CC133, and there was also segregation by farm within each host‐species. In contrast, as reported in the original paper (Grinberg et al. 2017), there was no segregation by host in any CC among human, canine and feline isolates. Although these results did not directly clarify the relative contributions of host‐adaptive selection and neutral drift to the diversification of the core genomes of ruminant *S. aureus* , they favour a model of neutral drift for three reasons. Firstly, neutral drift is reflected in the data by the core genome clustering by farm of bovine and ovine isolates, as such clustering cannot be attributed to a ‘species effect’. Second, the solitary bovine CC5 and CC15 isolates carried ‘ruminant’ virulomes, but co‐mingled with human isolates in the NJ trees, suggesting that adaptive selection of the core genome may not be required for the adaptation to ruminants. Lastly, a model of host‐adaptive selection would predict a segregation of the core genomes of human, canine, and feline isolates. However, our data show that in the absence of physical barriers for transmission within the household, such segregation is not observed.

Addressing the 2nd and 3rd benchmarks required an analysis of the accessory genomes. As for the core genome, the PCoA plot showed a primary differentiation of the isolates according to their CCs and secondarily by host species, except for human, canine and feline isolates that co‐mingled (Figure 3, upper plot). A different picture emerged when only the 197 virulence genes were plotted: while the virulomes of ruminant and human CC1 segregated, bovine and small ruminant isolates of CC8, CC133, CC15 and CC5 co‐mingled in the bidimensional space (lower plot). The co‐mingling of bovine and ovine isolates indicates an adaptation to ruminants sensu lato through convergent, adaptive homoplasy of the virulomes across CCs. This was subsequently supported by the gene‐by‐gene analysis, as bovine and small ruminant samples were rich in bovine‐adaptive LukF–LukM and lacked the PVL and IEC genes. The high prevalence of the LukM′F′ in small ruminant isolates suggests this factor may be biologically active also against ovine and caprine leukocytes, but to our knowledge this activity has not been studied. On the other hand, human, canine and feline samples were enriched with PVL and IEC genes, and lacked the LukM′F′. The high prevalence of the LukM′F′ in bovine strains is not novel. This leukocidin has been identified in about 80% of bovine isolates across lineages, more than a decade ago (Schlotter et al. 2012). 

Another point of difference between ruminant and human strains involved the resistome, with human/canine/feline *S. aureus* being 2.7 times more likely to possess one or more AMR genes than ruminant isolates. In particular, the *bla*Z gene was found in about 11% of bovine and 68% of human CC1, mirroring the contemporaneous level of phenotypic resistance to penicillin recorded in New Zealand for cattle and human *S. aureus* (Heffernan et al. 2015; Petrovski et al. 2015).

Our data contained only two CC1 outliers, namely the bovine B139 and the human H11, both having core and accessory genomes and virulomes matching the other host types. Such a scarce strain sharing was somehow surprising given the large size of the New Zealand dairy industry and the close physical interactions between farm personnel and cows on dairy farms. The marginal strain flow, combined with the verification of the three host‐adaptive benchmarks in virtually all ruminant isolates, are consistent with a ruminant‐specialist (rather than host‐adapted) MSSA sub‐population.

Lastly, the results of the metabolic profiling complement and expand existing data suggesting that bovine mastitis‐causing *S. aureus* metabolise lactose more effectively than human lineages. Far from being conclusive, the results for lactose and caproic acid suggest that ruminant‐adapted CC1 may be better equipped to grow in ruminant milk than non‐adapted strains.

In summary, we identified three fundamental genomic signatures of host adaptation in virtually all ruminant isolates across the clonal landscape, and very little strain flow across the human–ruminant interface. We report for the first time a ruminant‐adaptated CC1 clade, which together with previous reports of a poultry‐adapted ST5 clade (Lowder et al. 2009), challenges the generally held view of the CCs or STs as the taxonomic units defining the *S. aureus* adaptive success. The adaptation to ruminants does not appear to be species‐specific but *sensu lato*, and characterised by a convergent homoplasy of the virulome, with no evidence of an adaptive selection of the core genome. In New Zealand, ruminant *S. aureus* emerges as a host‐specialist sub‐population, clarifying why transmission of *S. aureus* between ruminants and humans has hardly ever been reported.

Host adaptation is a very complex phenomenon and many features remain unanswered. Hence, we deposited most of our draft genomes in the public domain and welcome others to use them.

## Author Contributions

**Jabin Nesaraj:** conceptualization, investigation, writing – review and editing, software, formal analysis, data curation, validation. **Alex Grinberg:** conceptualization, investigation, funding acquisition, writing – original draft, methodology, validation, visualization, formal analysis, project administration, supervision, resources. **Richard Laven:** conceptualization, funding acquisition, writing – review and editing, supervision. **Ryan Chanyi:** conceptualization, formal analysis, software, writing – review and editing, investigation. **Eric Altermann:** conceptualization, experimental design, data interpretation, reviewed draft. **Claudio Bandi:** conceptualization, resources, writing – review and editing. **Patrick J. Biggs:** conceptualization, investigation, writing – review and editing, software, supervision.

## Ethics Statement

The authors have nothing to report.

## Conflicts of Interest

The authors declare no conflicts of interest.

## Supporting information


Data S1.

## Data Availability

The genomes of the bovine, human, canine and feline *S. aureus* are available at the Sequence Read Archive of the US National Center for Biotechnology Information (https://www.ncbi.nlm.nih.gov/sra/); BioProjects PRJNA863911 and PRJNA391123.
