# Supplementary material for: The Host Adaptation of Staphylococcus aureus to Farmed Ruminants in New Zealand, With Special Reference to Clonal Complex 1
Source: Environ Microbiol Rep. 2025 May 6;17(3):e70087. doi: 10.1111/1758-2229.70087 (PMC12055752; doi:10.1111/1758-2229.70087)
Supplement: Supplementary file 1 — Data S1. [file EMI4-17-e70087-s001.docx]

**Supplementary Files**

**Supplementary File 1: MSSA Isolates**.

**Bovine isolates**: The New Zealand bovine *S. aureus* isolates used in this study were selected from a bank of isolates collected over a period of about 17 years by Dr Alex Grinberg, School of Veterinary Science, Massey University, New Zealand. They were donated by various collaborators and commercial diagnostic laboratories, and were stored at the Microbiology laboratory of the School. The isolates were obtained during three different time periods: 2002-03; 2012-13; and 2018-2019. Due to budget constrains, when selecting isolates from the collection, efforts were made to include isolates originating from a large number of farms and geographical regions.

2002-2003: The bovine isolates from 2002-2003 represented the almost complete set of isolates obtained from bovine milk specimens submitted to veterinary diagnostic laboratories from Hamilton and Palmerston North, New Zealand over a period of about 6 months, ending in February 2003. Laboratory staff were requested to submit multiple samples from the same farms but avoid duplicates from the same cow. Unique confidential identifiers for the farm of origin were supplied, without the geographical location of the farm. Other data supplied were the cow’s tag number and the laboratory location (Palmerston North or Hamilton). The isolates were phenotypically re-identified as *S. aureus* and stored in glycerol at -80◦C. A total of 116 isolates were available for this period. Two isolates originated from two-quarters of the same cow, so one was arbitrarily eliminated. Fifty nine farms were represented in this sample. Fifty-nine out of 115 isolates were selected, to represent one isolate per farm.

2012-2013: A total of 65 bovine isolates from 2012-13 were selected from a frozen collection of 134 isolates obtained from milk samples submitted to veterinary laboratories from Auckland, Hamilton, Palmerston North, Christchurch, and Dunedin, New Zealand. Data available included the name of the sourcing laboratory, the laboratory case number, date of collection and a descriptor of the geographical area of origin of the sample. No farm identifier was supplied. However, since the last three digits of the accession number represented the cow number, and the rest of the digits indicated the farm number, unique farm identifier could be assigned based on the accession number. For this study, one isolate was selected at random from each farm, for a total of 53 isolates. Subsequently, 12 additional isolates (one per cow) were selected from farms supplying multiple isolates, allowing also an analysis of multiple isolates per farm. Hence, a total of 65 isolates were used from this collection.

2017-2018: A total of 64 isolates from 2017-2018 were used. These isolates originated from two different sources. Fifty-one isolates were selected from a set of 79 isolates collected during a clinical trial of mastitis therapeutics performed on 11 dairy farms in central North Island. The isolates were donated for this study by the investigators. Isolates from this collection were included in the study based on the following criteria: firstly, from each farm, one isolate was selected at random from each treatment group (treated and control groups), for a total of 17 isolates (five farms had only isolates from either treatment or control groups). Secondly, 16 additional isolates from each farm, irrespective of groups, were selected randomly. The remaining 18 isolates were selected randomly from the post-treatment isolates obtained from non-cured cases. The remaining 13 isolates from 2017-18 were obtained from veterinary diagnostic laboratories in Hamilton and Palmerston North, New Zealand. The isolates arrived accompanied by the laboratory accession number. The last three digits of the accession number represented the cow number, and the rest of the digits indicated the farm number.

**Small Ruminant isolates**: Thirty sheep isolates originated from a field study assessing the prevalence of udder defects and bacterial pathogens in non-dairy-breed ewes performed on 11 sheep farms in the lower North Island in 2018-2019 (Ridler et al., 2021). Twelve of these isolates were obtained from sheep showing a variety of udder defects, 7 from sheep showing hard mastitis lesions, and six from sheep showing lumpy udder lesions. Five isolates originated from sheep with no lesions. The isolates came with unique identifiers for the farm and animal of origin. Two additional isolates were provided by a diagnostic laboratory, and were isolated in 2012 (from a goat) and 2018 (from a sheep). All the bovine and small ruminant isolates were stored in glycerol at -80°C.

**Human/canine/feline isolates:** For comparative analysis, this project used previously published genome sequences of 59 New Zealand human, canine, and feline *S. aureus* (Grinberg et al., 2017). These sequences were publicly available as reads in the Sequence Read Archive (SRA) of the US National Center for Biotechnology Information (NCBI) (Leinonen, Sugawara, & Shumway, 2011), as bio project number PRJNA391123, and were retrieved using the fastq-dump utility the sra-tool kit (Leinonen et al., 2011) with the ‘ --split-files’ option to convert the sra file to fastq file split into two paired-end reads.

**List of bovine isolates**

The identifiers of the 188 bovine *S*. *aureus* genomes. The ‘Unique Lab Identifier’ is the ‘Library Name’ identifier in Bioproject PRJNA863911 at the Sequence Read Archive (SRA) of the US National Center for Biotechnology Information (<https://www.ncbi.nlm.nih.gov/sra/>). The ‘Assigned names’ are the identifiers appearing in the paper’s graphs and are composed of five parts separated by an underscore. The first part denotes the isolate’s serial number, the second part is a unique farm identifier, the third indicates the year of collection, the fourth is the sequence type (ST), and the last is clonal complex (CC). For example, isolate B064_F51_Y13_ST1_CC1 is serial number B64, it was isolated from farm 51 in 2013, and it belongs to ST1, CC1..

| **Unique Lab Identifier** | **Assigned names** |  |
| --- | --- | --- |
| BM109 | B064_F51_Y13_ST1_CC1 |  |
| BM110 | B060_F48_Y13_ST1_CC1 |  |
| BM111 | B061_F48_Y13_ST1_CC1 |  |
| BM112 | B057_F45_Y13_ST1_CC1 |  |
| BM113 | B052_F41_Y13_ST1_CC1 |  |
| BM114 | B049_F38_Y13_ST78_CC78 |  |
| BM116 | B053_F06_Y13_ST1_CC1 |  |
| BM117 | B054_F42_Y13_ST1_CC1 |  |
| BM118 | B045_F34_Y13_ST1_CC1 |  |
| BM119 | B059_F47_Y13_ST1_CC1 |  |
| BM123 | B047_F36_Y13_ST97_CC97 |  |
| BM124 | B043_F33_Y13_ST1_CC1 |  |
| BM126 | B044_F33_Y13_ST1_CC1 |  |
| BM128 | B040_F30_Y13_ST4551_CC1 |  |
| BM129 | B036_F137_Y13_ST1_CC1 |  |
| BM130 | B037_F28_Y13_ST1_CC1 |  |
| BM131 | B063_F50_Y13_ST1_CC1 |  |
| BM132 | B041_F31_Y13_ST1_CC1 |  |
| BM133 | B051_F40_Y13_ST1_CC1 |  |
| BM134 | B038_F29_Y13_ST1_CC1 |  |
| BM136 | B039_F29_Y13_ST1_CC1 |  |
| BM137 | B046_F35_Y13_ST97_CC97 |  |
| BM33 | B001_F01_Y13_ST6143_CC8 |  |
| BM34 | B002_F01_Y13_ST6143_CC8 |  |
| BM35 | B003_F02_Y13_ST1_CC1 |  |
| BM36 | B004_F03_Y13_ST1_CC1 |  |
| BM37 | B005_F04_Y13_ST97_CC97 |  |
| BM38 | B006_F05_Y13_ST1_CC1 |  |
| BM39 | B007_F02_Y13_ST1_CC1 |  |
| BM40 | B008_F05_Y13_ST1_CC1 |  |
| BM41 | B009_F07_Y13_ST1_CC1 |  |
| BM42 | B010_F07_Y13_ST1_CC1 |  |
| BM43 | B011_F03_Y13_ST1_CC1 |  |
| BM45 | B012_F08_Y13_ST1_CC1 |  |
| BM46 | B013_F09_Y13_ST6143_CC8 | |
| BM49 | B014_F10_Y13_ST1_CC1 | |
| BM51 | B016_F12_Y13_ST1_CC1 | |
| BM52 | B017_F134_Y13_ST1_CC1 | |
| BM53 | B018_F13_Y13_ST1_CC1 | |
| BM54 | B019_F14_Y13_ST97_CC97 | |
| BM55 | B020_F15_Y13_ST1_CC1 | |
| BM57 | B021_F16_Y13_ST1_CC1 | |
| BM58 | B022_F17_Y13_ST1_CC1 | |
| BM59 | B023_F18_Y13_ST199_CC15 | |
| BM60 | B024_F19_Y13_ST1_CC1 | |
| BM61 | B025_F13_Y13_ST97_CC97 | |
| BM63 | B026_F20_Y13_ST97_CC97 | |
| BM64 | B027_F21_Y13_ST1247_CC133 | |
| BM65 | B028_F22_Y13_ST1_CC1 | |
| BM66 | B029_F23_Y13_ST97_CC97 | |
| BM67 | B030_F24_Y13_ST1_CC1 | |
| BM69 | B031_F25_Y13_ST8_CC8 | |
| BM71 | B032_F26_Y13_ST1_CC1 | |
| BM72 | B033_F08_Y13_ST1_CC1 | |
| BM73 | B034_F19_Y13_ST97_CC97 | |
| BM75 | B035_F27_Y13_ST1_CC1 | |
| BM76 | B058_F46_Y13_ST1_CC1 | |
| BM77 | B055_F43_Y13_ST1_CC1 | |
| BM81 | B050_F39_Y13_ST1_CC1 | |
| BM85 | B062_F49_Y13_ST97_CC97 | |
| BM87 | B042_F32_Y13_ST1_CC1 | |
| BM89 | B048_F37_Y13_ST1_CC1 | |
| BM90 | B056_F44_Y13_ST1_CC1 | |
| BM92 | B065_F52_Y13_ST1_CC1 | |
| H01 | B066_F54_Y18_ST1_CC1 | |
| H02 | B067_F55_Y18_ST97_CC97 | |
| H03 | B068_F56_Y18_ST151_CC151 | |
| H04 | B069_F55_Y18_ST97_CC97 | |
| H05 | B070_F54_Y18_ST1_CC1 | |
| H06 | B071_F57_Y18_ST151_CC151 | |
| H07 | B072_F58_Y18_ST5367_CC5367 | |
| H08 | B076_F59_Y18_ST1_CC1 | |
| H09 | B073_F55_Y18_ST97_CC97 | |
| H10 | B077_F60_Y18_ST97_CC97 | |
| H11 | B074_F55_Y18_ST97_CC97 | |
| H12 | B075_F58_Y18_ST5367_CC5367 | |
| H13 | B078_F61_Y18_ST1_CC1 | |
| S010 | B083_F63_Y18_ST1_CC1 | |
| S105 | B110_F62_Y18_ST6160_CC97 | |
| S107 | B116_F62_Y18_ST6160_CC97 | |
| S108 | B117_F62_Y18_ST6160_CC97 | |
| S109 | B118_F62_Y18_ST6160_CC97 | |
| S110 | B120_F62_Y18_ST6160_CC97 | |
| S117 | B126_F73_Y18_ST1_CC1 | |
| S118 | B125_F72_Y18_ST1_CC1 | |
| S138 | B122_F71_Y18_ST1_CC1 | |
| S142 | B124_F69_Y18_ST1_CC1 | |
| S144 | B123_F69_Y18_ST1_CC1 | |
| S015 | B084_F64_Y18_ST1_CC1 | |
| S153 | B111_F70_Y18_ST1_CC1 | |
| S154 | B112_F70_Y18_ST1_CC1 | |
| S155 | B113_F70_Y18_ST1_CC1 | |
| S156 | B119_F62_Y18_ST1_CC1 | |
| S159 | B114_F66_Y18_ST1_CC1 | |
| S166 | B121_F62_Y18_ST1_CC1 | |
| S171 | B115_F65_Y18_ST1_CC1 | |
| S181 | B127_F63_Y18_ST1_CC1 | |
| S182 | B128_F63_Y18_ST1_CC1 | |
| S183 | B129_F63_Y18_ST1_CC1 | |
| S002 | B079_F62_Y18_ST6160_CC97 | |
| S021 | B085_F65_Y18_ST1_CC1 | |
| S024 | B086_F66_Y18_ST1_CC1 | |
| S027 | B087_F65_Y18_ST1_CC1 | |
| S028 | B088_F65_Y18_ST1_CC1 | |
| S021 | B089_F65_Y18_ST1_CC1 | |
| S024 | B090_F67_Y18_ST6141_CC1 | |
| S025 | B091_F67_Y18_ST6141_CC1 | |
| S019 | B092_F65_Y18_ST1_CC1 | |
| S020 | B093_F65_Y18_ST1_CC1 | |
| S021 | B094_F65_Y18_ST1_CC1 | |
| S023 | B095_F68_Y18_ST1_CC1 | |
| S024 | B096_F68_Y18_ST1_CC1 | |
| S028 | B097_F63_Y18_ST1_CC1 | |
| S006 | B080_F62_Y18_ST6160_CC97 | |
| S015 | B098_F69_Y18_ST1_CC1 | |
| S026 | B099_F69_Y18_ST1_CC1 | |
| S007 | B081_F62_Y18_ST6160_CC97 | |
| S029 | B100_F64_Y18_ST1_CC1 | |
| S020 | B101_F64_Y18_ST1_CC1 | |
| S014 | B102_F65_Y18_ST1_CC1 | |
| S026 | B103_F65_Y18_ST6161_CC1 | |
| S027 | B104_F65_Y18_ST1_CC1 | |
| S028 | B105_F65_Y18_ST1_CC1 | |
| S029 | B106_F65_Y18_ST1_CC1 | |
| S029 | B082_F63_Y18_ST1_CC1 | |
| S026 | B107_F70_Y18_ST6140_CC1 | |
| S027 | B108_F62_Y18_ST1_CC1 | |
| S028 | B109_F62_Y18_ST1_CC1 | |
| ST116 | B136_F80_Y02_ST1_CC1 | |
| ST117 | B132_F76_Y02_ST5_CC5 | |
| ST119 | B137_F81_Y02_ST508_CC45 | |
| ST120 | B133_F77_Y02_ST1_CC1 | |
| ST130 | B134_F78_Y02_ST705_CC151 | |
| ST132 | B135_F79_Y02_ST8_CC8 | |
| ST135 | B148_F92_Y02_ST1_CC1 | |
| ST136 | B131_F75_Y02_ST97_CC97 | |
| ST137 | B138_F82_Y02_ST8_CC8 | |
| ST143 | B139_F83_Y02_ST1_CC1 | |
| ST144 | B158_F102_Y02_ST6162_CC97 | |
| ST147 | B142_F86_Y02_ST1_CC1 | |
| ST150 | B149_F93_Y02_ST6163_CC1 | |
| ST153 | B147_F91_Y02_ST1_CC1 | |
| ST154 | B159_F103_Y02_ST1_CC1 | |
| ST155 | B160_F104_Y02_ST1_CC1 | |
| ST156 | B161_F105_Y02_ST1_CC1 | |
| ST157 | B143_F87_Y02_ST1_CC1 | |
| ST159 | B162_F106_Y02_ST1_CC1 | |
| ST160 | B163_F107_Y02_ST1_CC1 | |
| ST163 | B164_F108_Y02_ST71_CC97 | |
| ST164 | B150_F94_Y02_ST1_CC1 | |
| ST166 | B151_F95_Y02_ST151_CC151 | |
| ST167 | B165_F109_Y02_ST1_CC1 | |
| ST168 | B154_F98_Y02_ST1_CC1 | |
| ST169 | B166_F111_Y02_ST1_CC1 | |
| ST173 | B144_F88_Y02_ST1_CC1 | |
| ST174 | B167_F112_Y02_ST1_CC1 | |
| ST176 | B168_F113_Y02_ST1_CC1 | |
| ST177 | B155_F99_Y02_ST1_CC1 | |
| ST180 | B153_F97_Y02_ST1_CC1 | |
| ST181 | B169_F114_Y02_ST1_CC1 | |
| ST182 | B170_F115_Y02_ST1_CC1 | |
| ST185 | B152_F96_Y02_ST1_CC1 | |
| ST187 | B171_F116_Y02_ST1_CC1 | |
| ST188 | B172_F117_Y02_ST1_CC1 | |
| ST189 | B173_F118_Y02_ST1_CC1 | |
| ST196 | B174_F119_Y02_ST1_CC1 | |
| ST198 | B177_F122_Y02_ST1_CC1 | |
| ST199 | B141_F85_Y02_ST97_CC97 |  |
| ST200 | B178_F123_Y02_ST1_CC1 |  |
| ST202 | B145_F89_Y02_ST151_CC151 |  |
| ST203 | B179_F124_Y02_ST151_CC151 |  |
| ST204 | B180_F125_Y02_ST6164_CC97 |  |
| ST205 | B157_F101_Y02_ST1_CC1 |  |
| ST206 | B140_F84_Y02_ST1_CC1 |  |
| ST208 | B181_F126_Y02_ST1_CC1 |  |
| ST209 | B182_F127_Y02_ST1_CC1 |  |
| ST211 | B175_F120_Y02_ST1_CC1 |  |
| ST212 | B176_F121_Y02_ST6165_CC133 |  |
| ST213 | B156_F100_Y02_ST1_CC1 |  |
| ST215 | B183_F128_Y02_ST1_CC1 |  |
| ST216 | B184_F129_Y02_ST151_CC151 |  |
| ST217 | B185_F130_Y02_ST1_CC1 |  |
| ST218 | B186_F131_Y02_ST1_CC1 |  |
| ST219 | B187_F132_Y02_ST1_CC1 |  |
| ST220 | B146_F90_Y02_ST6140_CC1 |  |
| ST222 | B188_F133_Y02_ST1_CC1 |  |
| ST99 | B130_F74_Y02_ST1_CC1 |  |

**List of small ruminant isolates (these genomes were not available publicly at the time of this publication):**

| **Unique** | **Geographical origin (if** | **Assigned farm** |
| --- | --- | --- |
| **Lab Identifier** | **available)** | **number** |
| BM50 | Waitara | F11 |
| GM1 |  | F53 |
| 420R | Central North Island | F01 |
| 2JR | Central North Island | F02 |
| 5JR | Central North Island | F02 |
| 6JR | Central North Island | F02 |
| 4101R | Central North Island | F03 |
| 17 | Central North Island | F03 |
| 11 | Central North Island | F03 |
| 4305R | Central North Island | F03 |
| 16BT-A | Central North Island | F04 |
| 15PW | Central North Island | F05 |
| 17BT | Central North Island | F04 |
| 11MR | Central North Island | F06 |
| 3JR | Central North Island | F02 |
| 12GS | Central North Island | F07 |
| 15IH-B | Central North Island | F08 |
| 1IH | Central North Island | F08 |
| NP2 | Central North Island | F09 |
| NP1 | Central North Island | F09 |
| 15IH-A | Central North Island | F08 |
| NP15 | Central North Island | F09 |
| 2IH | Central North Island | F08 |
| 5 | Central North Island | F03 |
| 2 | Central North Island | F03 |
| 3 | Central North Island | F03 |
| NP3 | Central North Island | F09 |
| 3JB | Central North Island | F10 |
| 5JB | Central North Island | F10 |

**Human, canine and feline genomes (genomes available as Bioproject PRJNA391123 at the Sequence Read Archive (SRA) of the US National Center for Biotechnology Information (**[**https://www.ncbi.nlm.nih.gov/sra/**](https://www.ncbi.nlm.nih.gov/sra/)**).**

| SRA Accession number | Original unique identifier | Source of isolate |
| --- | --- | --- |
| SRR5714651 | H44 | Canine clinical |
| SRR5714659 | H31 | Canine clinical |
| SRR5714675 | H57 | Canine clinical |
| SRR5714690 | H3 | Canine clinical |
| SRR5714693 | H8 | Canine clinical |
| SRR5714696 | H9 | Canine clinical |
| SRR5714699 | H21 | Canine clinical |
| SRR5714704 | H26 | Canine clinical |
| SRR5714706 | H30 | Canine clinical |
| SRR5714653 | H46 | Canine colonisation |
| SRR5714654 | H47 | Canine colonisation |
| SRR5714655 | H48 | Canine colonisation |
| SRR5714658 | H32 | Canine colonisation |
| SRR5714664 | H33 | Canine colonisation |
| SRR5714684 | H34 | Canine colonisation |
| SRR5714694 | H7 | Canine colonisation |
| SRR5714700 | H22 | Canine colonisation |
| SRR5714702 | H28 | Canine colonisation |
| SRR5714705 | H29 | Canine colonisation |
| SRR5714676 | H56 | Feline clinical |
| SRR5714689 | H4 | Feline clinical |
| SRR5714691 | H6 | Feline clinical |
| SRR5714692 | H5 | Feline clinical |
| SRR5714652 | H45 | Feline colonisation |
| SRR5714695 | H10 | Feline colonisation |
| SRR5714648 | H41 | Human clinical |
| SRR5714650 | H43 | Human clinical |
| SRR5714660 | H20 | Human clinical |
| SRR5714661 | H19 | Human clinical |
| SRR5714670 | H18 | Human clinical |
| SRR5714671 | H17 | Human clinical |
| SRR5714677 | H55 | Human clinical |
| SRR5714678 | H54 | Human clinical |
| SRR5714679 | H53 | Human clinical |
| SRR5714680 | H52 | Human clinical |
| SRR5714681 | H51 | Human clinical |
| SRR5714683 | H59 | Human clinical |
| SRR5714687 | H2 | Human clinical |
| SRR5714703 | H25 | Human clinical |
| SRR5714649 | H42 | Human colonisation |
| SRR5714656 | H49 | Human colonisation |
| SRR5714657 | H50 | Human colonisation |
| SRR5714662 | H36 | Human colonisation |
| SRR5714663 | H35 | Human colonisation |
| SRR5714665 | H37 | Human colonisation |
| SRR5714666 | H14 | Human colonisation |
| SRR5714667 | H13 | Human colonisation |
| SRR5714668 | H12 | Human colonisation |
| SRR5714669 | H11 | Human colonisation |

| SRA Accession number | Original unique identifier | Source of isolate |
| --- | --- | --- |
| SRR5714672 | H16 | Human colonisation |
| SRR5714673 | H15 | Human colonisation |
| SRR5714674 | H58 | Human colonisation |
| SRR5714682 | H38 | Human colonisation |
| SRR5714685 | H40 | Human colonisation |
| SRR5714686 | H39 | Human colonisation |
| SRR5714688 | H1 | Human colonisation |
| SRR5714697 | H23 | Human colonisation |
| SRR5714698 | H24 | Human colonisation |
| SRR5714701 | H27 | Human colonisation |

**Supplementary File 2:**

**Omnilog Method:**

For each strain, a bacterial suspension was prepared in 10 mL 1x inoculating fluid IF-0a (Biolog, USA) until a 81% transmittance was measured using a turbidiometer. A 12x additive solution containing 24 mM MgCl_2_, 12 mM CaCl_2_, 0.3 mM arginine hydrochloride, 0.6 mM monosodium glutamate, 0.001% (w/v) yeast extract, 0.15 mM L-cystine, 0.3 mM uridine 5’-monophosphate was prepared. For each strain, 2mL additive, 0.24 mL Redox Dye H (Biolog, USA) and 1.76 mL of bacterial suspension were added to 20mL 1.2x IF-0a and 100µL was transferred to each well of a PM1 and PM2 plates. Isolates were run in duplicate, with one replicate per plate, 95 carbon sources, and a blank without the carbon source. Plates were incubated as a kinetic run in the Omnilog® Phenotype Microarray system at 37°C with optical measurements taken at 15-minute intervals for 48h. Data were collected using the Biolog Kinetic and Parametric Analysis software (v1.7.1.58) and analysed using the OPM package (v1.3.36) in R (Vaas et al. 2012; Vaas et al. 2013).

**Supplementary File 3: Spa types**

| **Spa-type** | **Host species** | **Associated CCs** | **N (%)** |
| --- | --- | --- | --- |
| t114 | Bovine | CC1(ST1, ST6140, ST6161, ST4551) | 107 (38.6%) |
| t127 | Bovine, canine, Feline, Human | CC1 (ST1) | 19 (6.9%) |
| t189 | Canine, Feline, Human | CC188(ST188, ST188v) | 15 (5.4%) |
| t524 | Bovine | CC97(ST6160, ST71) | 9 (3.2%) |
| t922 | Bovine | CC1(ST1, ST6163) | 7 (2.5%) |
| t529 | Bovine | CC151(ST151, ST705) | 7 (2.5%) |
| t059 | Sheep | CC8 (ST8) | 5 (1.8%) |
| t1265 | Canine, Human | CC5 (ST5) | 5 (1.8%) |
| tnew8 (14-23-17-362-13-22-22) | Bovine, Sheep | CC5367 (ST5367) | 5 (1.8%) |
| tnew10 (03-12-21-17-23-13-17-17-23-24) | Sheep | CC133(ST6157, ST6138, ST133) | 5 (1.8%) |
| t1784 | Bovine | CC1 (ST1) | 4 (1.4%) |
| t693 | Bovine | CC1(ST1) | 4 (1.4%) |
| t118 | Sheep | CC8 (ST8) | 3 (1.1%) |
| t179 | Canine, Human | CC5(ST5) | 3 (1.1%) |
| t084 | Bovine, Human | CC15 (ST199, ST15) | 3 (1.1%) |
| t2699 | Bovine | CC1(ST1) | 3 (1.1%) |
| t211 | Bovine | CC8 (ST6143) | 3 (1.1%) |
| t008 | Bovine | CC8 (ST8) | 2 (0.7%) |
| t019 | Human | CC30 (ST30) | 2 (0.7%) |
| t16350 | Bovine | CC97 (ST97) | 2 (0.7%) |
| t17282 | Bovine | CC97 (ST97) | 2 (0.7%) |
| t2207 | Canine | CC1(ST1) | 2 (0.7%) |
| t267 | Bovine | CC97 (ST97) | 2 (0.7%) |
| t3047 | Sheep | CC133(ST133, ST6139) | 2 (0.7%) |
| t3380 | Bovine | CC97(ST97) | 2 (0.7%) |
| t4735 | Sheep | CC133(ST133, ST701) | 2 (0.7%) |
| t6980 | Bovine | CC1(ST1, ST6140) | 2 (0.7%) |
| tnew1 (07-16-02-13) | Bovine | CC1 (ST6141) | 2 (0.7%) |
| t002 | Human | CC5 (ST5, ST835) | 2 (0.7%) |
| t010 | Human | CC5 (ST5) | 1 (0.4%) |
| t012 | Human | CC30 (ST30v) | 1 (0.4%) |
| t015 | Bovine | CC45 (ST508) | 1 (0.4%) |
| t062 | Bovine | CC5(ST5) | 1 (0.4%) |
| t089 | Canine | CC30 (ST34) | 1 (0.4%) |
| t1166 | Bovine | CC133 (ST1247) | 1 (0.4%) |
| t1407 | Bovine | CC1(ST1) | 1 (0.4%) |
| t1509 | Canine | CC15(ST582) | 1 (0.4%) |
| t15807 | Sheep | CC133(ST133) | 1 (0.4%) |
| t16141 | Human | CC1 (ST1) | 1 (0.4%) |
| t177 | Sheep | CC1 (ST3615) | 1 (0.4%) |
| t186 | Bovine | CC78(ST78) | 1 (0.4%) |
| t1931 | Bovine | CC1(ST1) | 1 (0.4%) |
| t2246 | Bovine | CC1(ST1) | 1 (0.4%) |
| t2421 | Bovine | CC97 (ST97) | 1 (0.4%) |
| t2802 | Bovine | CC97(ST97) | 1 (0.4%) |
| t2868 | Human | CC30(ST30) | 1 (0.4%) |
| t386 | Bovine | CC1(ST1) | 1 (0.4%) |
| t4453 | Sheep | CC1640(ST1640) | 1 (0.4%) |
| t4540 | Bovine | CC1(ST1) | 1 (0.4%) |
| t4558 | Feline | CC188(ST188) | 1 (0.4%) |
| t4601 | Canine | CC1(ST1) | 1 (0.4%) |
| t4682 | Bovine | CC97(ST6164) | 1 (0.4%) |
| t5150 | Human | CC5(ST5) | 1 (0.4%) |
| t5213 | Canine | CC5(ST5) | 1 (0.4%) |
| t5402 | Canine | CC5(ST1259) | 1 (0.4%) |
| t6690 | Human | CC5(ST5) | 1 (0.4%) |
| t7310 | Sheep | CC133(ST133) | 1 (0.4%) |
| t7623 | Canine | CC30(ST30) | 1 (0.4%) |
| t865 | Bovine | CC97(ST6162) | 1 (0.4%) |
| t8914 | Canine | CC188(ST188) | 1 (0.4%) |
| t1414 | Canine | CC30(ST30) | 1 (0.4%) |
| t17906 | Bovine | CC133(ST6165) | 1 (0.4%) |
| t2271 | Canine | CC30 (ST39) | 1 (0.4%) |
| tnew11 (03-16-05-17-23-13-17-17-17-17-17-23-24) | Sheep | CC133 (ST6137) | 1 (0.4%) |
| tnew12 (03-16-05-17-23-13-17-17-17-17-23-24) | Sheep | CC133(ST133) | 1 (0.4%) |
| tnew13 (03-16-12-21-17-23-13-17-17-23-31-24) | Sheep | CC133(ST6166) | 1 (0.4%) |
| tnew2 (07-23-12-05-17-02-17-34-34-34-34-33-34) | Bovine | CC97(ST97) | 1 (0.4%) |
| tnew3 (07-23-12-05-17-34-34-13-33-34) | Bovine | CC97(ST97) | 1 (0.4%) |
| tnew4 (07-23-12-05-22-34-13-34-13-33-34) | Bovine | CC97 (ST97) | 1 (0.4%) |
| tnew5 (11-19-12-21-17-34-24-34-24-34-22-33-25) | Bovine | CC8 (ST8) | 1 (0.4%) |
| tnew14 (121-21-16-82-24-17-17-17-17-17) | Sheep | CC1640 (ST1640) | 1 (0.4%) |
| tnew6 (14-12-05-17-34-34-13-33-34) | Bovine | CC97 (ST97) | 1 (0.4%) |
| tnew7 (14-12-05-17-34-34-34-13-33-34) | Bovine | CC97 (ST97) | 1 (0.4%) |
| tnew9 (16-34-33-13) | Bovine | CC1 (ST1) | 1 (0.4%) |
| tnew15 (26-23-12-21-17-34-34-25-17) | Sheep | CC692 (ST692) | 1 (0.4%) |
| tnew16 (591-16-34-17-82-24-17-17-17-17-17) | Sheep | CC1640 (ST1640) | 1 (0.4%) |

**Supplementary File 4: Host adaptive gene distribution: Number of isolates carrying each host adaptive factor, stratified by CC and host species**

| **Host** | **CC(Total)** | **scn** | **sak** | **chp** | **sea** | **lukF_PV** | **lukS_PV** | **lukF'** | **lukM** | **icaA** | **icaB** | **icaC** | **icaD** | **icaR** | **tsst** |
| --- | --- | --- | --- | --- | --- | --- | --- | --- | --- | --- | --- | --- | --- | --- | --- |
| Bovine | CC1(100) | 1(1%) | 1(1%) | 0(0%) | 1(1%) | 1(1%) | 1(1%) | 80(80%) | 79(79%) | 17(17%) | 99(99%) | 100(100%) | 15(15%) | 100(100%) | 0(0%) |
| Bovine | CC133(2) | 0(0%) | 0(0%) | 0(0%) | 0(0%) | 0(0%) | 0(0%) | 0(0%) | 0(0%) | 2(100%) | 2(100%) | 2(100%) | 2(100%) | 2(100%) | 0(0%) |
| Bovine | CC15(1) | 1(100%) | 1(1%) | 1(100%) | 0(0%) | 0(0%) | 0(0%) | 1(100%) | 1(100%) | 1(100%) | 1(100%) | 0(0%) | 1(100%) | 1(0%) | 0(0%) |
| Bovine | CC151(7) | 0(0%) | 0(0%) | 0(0%) | 0(0%) | 0(0%) | 0(0%) | 7(100%) | 7(100%) | 7(100%) | 7(100%) | 7(100%) | 7(100%) | 7(100%) | 1(14.3%) |
| Bovine | CC45(1) | 1(100%) | 1(1%) | 1(100%) | 0(0%) | 0(0%) | 0(0%) | 0(0%) | 0(0%) | 1(100%) | 1(100%) | 1(100%) | 1(100%) | 1(100%) | 0(0%) |
| Bovine | CC5(1) | 0(0%) | 0(0%) | 0(0%) | 0(0%) | 0(0%) | 0(0%) | 0(0%) | 0(0%) | 1(100%) | 1(100%) | 1(100%) | 1(100%) | 1(100%) | 0(0%) |
| Bovine | CC5367(1) | 0(0%) | 0(0%) | 0(0%) | 0(0%) | 0(0%) | 0(0%) | 0(0%) | 0(0%) | 1(100%) | 1(100%) | 1(100%) | 1(100%) | 1(100%) | 0(0%) |
| Bovine | CC78(1) | 1(100%) | 1(1%) | 0(0%) | 0(0%) | 0(0%) | 0(0%) | 0(0%) | 0(0%) | 1(100%) | 1(100%) | 1(100%) | 1(100%) | 1(100%) | 0(0%) |
| Bovine | CC8(5) | 0(0%) | 0(0%) | 0(0%) | 0(0%) | 0(0%) | 0(0%) | 0(0%) | 0(0%) | 5(100%) | 5(100%) | 5(100%) | 5(100%) | 5(100%) | 0(0%) |
| Bovine | CC97(17) | 0(0%) | 0(0%) | 0(0%) | 0(0%) | 0(0%) | 0(0%) | 2(11%) | 2(11%) | 15(88%) | 8(47%) | 15(88%) | 15(88%) | 15(88%) | 0(0%) |
| Canine | CC1(5) | 4(80%) | 5(5%) | 2(40%) | 2(40%) | 2(40%) | 2(40%) | 0(0%) | 0(0%) | 5(100%) | 5(100%) | 4(80%) | 5(100%) | 2(80%) | 0(0%) |
| Canine | CC15(1) | 1(100%) | 0(0%) | 0(0%) | 0(0%) | 0(0%) | 0(0%) | 0(0%) | 0(0%) | 1(100%) | 0(0%) | 1(100%) | 1(100%) | 1(100%) | 0(0%) |
| Canine | CC188(3) | 1(33%) | 3(3%) | 3(100%) | 0(0%) | 0(0%) | 0(0%) | 0(0%) | 0(0%) | 2(66%) | 3(100%) | 3(100%) | 3(100%) | 2(100%) | 0(0%) |
| Canine | CC30(4) | 3(75%) | 4(4%) | 2(50%) | 2(50%) | 1(25%) | 1(25%) | 0(0%) | 0(0%) | 4(100%) | 3(75%) | 4(100%) | 4(100%) | 2(100%) | 2(50%) |
| Canine | CC5(6) | 4(66%) | 3(3%) | 4(66%) | 0(0%) | 0(0%) | 0(0%) | 0(0%) | 0(0%) | 6(100%) | 3(50%) | 4(66%) | 6(100%) | 4(66%) | 0(0%) |
| Feline | CC1(2) | 1(50%) | 1(1%) | 1(50%) | 0(0%) | 1(50%) | 1(50%) | 0(0%) | 0(0%) | 2(100%) | 2(100%) | 2(100%) | 2(100%) | 2(100%) | 0(0%) |
| Feline | CC188(4) | 4(100%) | 4(4%) | 2(50%) | 0(0%) | 0(0%) | 0(0%) | 0(0%) | 0(0%) | 4(100%) | 3(75%) | 2(50%) | 4(100%) | 3(50%) | 0(0%) |
| Human | CC1(9) | 6(66%) | 8(8%) | 1(11%) | 8(88%) | 1(11%) | 1(11%) | 0(0%) | 0(0%) | 9(100%) | 9(100%) | 8(88%) | 9(100%) | 7(88%) | 0(0%) |
| Human | CC15(2) | 2(100%) | 0(0%) | 2(100%) | 0(0%) | 0(0%) | 0(0%) | 0(0%) | 0(0%) | 2(100%) | 0(0%) | 2(100%) | 2(100%) | 1(100%) | 0(0%) |
| Human | CC188(10) | 6(60%) | 10(10%) | 7(70%) | 0(0%) | 0(0%) | 0(0%) | 0(0%) | 0(0%) | 9(90%) | 10(100%) | 10(100%) | 10(100%) | 9(100%) | 0(0%) |
| Human | CC30(3) | 2(66%) | 2(2%) | 1(33%) | 1(33%) | 1(33%) | 1(33%) | 0(0%) | 0(0%) | 3(100%) | 2(66%) | 3(100%) | 3(100%) | 2(100%) | 2(66%) |
| Human | CC5(9) | 5(55%) | 9(9%) | 7(77%) | 0(0%) | 0(0%) | 0(0%) | 0(0%) | 0(0%) | 9(100%) | 8(88%) | 6(66%) | 8(88%) | 6(66%) | 0(0%) |
| Small ruminant | CC1(1) | 0(0%) | 0(0%) | 0(0%) | 0(0%) | 0(0%) | 0(0%) | 0(0%) | 0(0%) | 1(100%) | 1(100%) | 1(100%) | 1(100%) | 1(100%) | 0(0%) |
| Small ruminant | CC133(9) | 0(0%) | 0(0%) | 0(0%) | 0(0%) | 0(0%) | 0(0%) | 9(100%) | 9(100%) | 9(100%) | 9(100%) | 9(100%) | 9(100%) | 8(100%) | 9(100%) |
| Small ruminant | CC1640(2) | 0(0%) | 0(0%) | 0(0%) | 0(0%) | 0(0%) | 0(0%) | 0(0%) | 0(0%) | 2(100%) | 2(100%) | 2(100%) | 2(100%) | 2(100%) | 0(0%) |
| Small ruminant | CC5367(1) | 0(0%) | 0(0%) | 0(0%) | 0(0%) | 0(0%) | 0(0%) | 0(0%) | 0(0%) | 1(100%) | 0(0%) | 1(100%) | 1(100%) | 1(100%) | 0(0%) |
| Small ruminant | CC692(1) | 0(0%) | 0(0%) | 0(0%) | 0(0%) | 0(0%) | 0(0%) | 0(0%) | 0(0%) | 1(100%) | 1(100%) | 1(100%) | 1(100%) | 1(100%) | 0(0%) |
| Small ruminant | CC8(3) | 0(0%) | 0(0%) | 0(0%) | 0(0%) | 0(0%) | 0(0%) | 2(66%) | 3(100%) | 3(100%) | 3(100%) | 3(100%) | 3(100%) | 3(100%) | 0(0%) |

**Supplementary File 5: Resistome Distribution**

| Resistance genes | Number of isolates (n=212) | Host | Clonal complex |
| --- | --- | --- | --- |
| *ant* | 1 (0.5%) | Human (1/59; 1.7%) | CC30(1/8; 12.5%) |
| *aph* | 2 (0.9%) | Human (2/59; 3.4%) | CC1(2/117; 1.7%) |
| *blaZ* | 76 (35.8%) | Bovine (34/136; 25%) Human, canine and feline (40/59; 67.8%), Small-ruminants (2/17; 11.8%) | CC1(26/117; 22.2%), CC133(1/11; 9.1%), CC15(3/4; 75%), CC188(9/17; 52.9%), CC30(5/8; 62.5%), CC45(1/1; 100%), CC5(12/16; 75%), CC78(1/1; 100%), CC8 (4/8; 50%), CC97(14/17; 82.5%) |
| *ermA* | 1 (0.5%) | Human (1/59; 1.7%) | CC30 (1/8; 12.5%) |
| *ermB* | 1 (0.5%) | Human (1/59; 1.7%) | CC1 (1/117; 0.9%) |
| *ermC* | 2 (0.9%) | Bovine (2/136; 1.5%) | CC1 (2/117; 1.7%) |
| *fusC* | 8 (3.8%) | Bovine (3/136; 2.2%), Human and canine (5/59; 8.5%) | CC1(2/117; 1.7%), CC188(1/17; 5.9%), CC5(3/16; 18.8%), CC8(2/8; 25%) |
| *str* | 2 (0.9%) | Bovine (2/136; 1.5%) | CC1 (2/117; 1.7%) |
| No resistance genes | 132 (62.3%) | Bovine (100/136; 73.5%), Human, canine and feline (17/59; 28.8%), small ruminants (15/17; 88.2%) | CC1 (89/117; 76.1%), CC133 (10/11; 90.9%), CC15 (1/4; 25%), CC151 (7/7; 100%), CC1640 (2/2; 100%), CC188(7/17; 41.2%), CC30 (3/8; 37.5%), CC5 (3/16; 18.8%), CC5367 (2/2; 100%), CC692(1/1; 100%), CC8 (4/8; 50%), CC97 (3/17; 17.6%) |
